# Application of the WHO Keys of Safer Food to Improve Food Handling Practices of Food Vendors in a Poor Resource Community in Ghana

**DOI:** 10.3390/ijerph6112833

**Published:** 2009-11-13

**Authors:** Eric S. Donkor, Boniface B. Kayang, Jonathan Quaye, Moses L. Akyeh

**Affiliations:** 1 Department of Microbiology, University of Ghana Medical School, Accra, Ghana; 2 London School of Hygiene and Tropical Medicine, University of London, London, WC1E 7HT, UK; 3 Department of Animal Science, School of Agriculture, University of Ghana, Accra, Ghana; E-Mails: bbkayang@hotmail.com (B.B.K.); quayenona@yahoo.com (J.Q.); 4 Bacteriology Unit, Noguchi Memorial Institute for Medical Research, University of Ghana, Accra, Ghana; E-Mail: LAkyeh@noguchi.mimcom.org

**Keywords:** food safety, vendors, Ghana

## Abstract

Data was collected from food vendors in a poor resource community in Ghana, which showed that the vendors constituted an important source of oro-faecal transmission. Following this, the WHO five keys of safer food were utilized in an evidence based training programme for the vendors to improve their food handling practices. Impact assessment of the food safety training showed that 67.6% of the vendors had acquired some knowledge from the workshop and were putting it into practice. Lack of food safety equipment was a major hinderance to behavioral change among the vendors as far food handling practices are concerned.

## 1. Introduction

In many developing countries, street food or ready-to-eat food vendors are an important component of the food supply chain. Street food satisfies a vital need of the urban population by being reasonably priced and conveniently available, and some segments of the population depend entirely on it. Food safety is a major concern with street foods as these foods are generally prepared and sold under unhygienic conditions, with limited access to safe water, sanitary services, or garbage disposal facilities [1,2]. Hence street foods pose a high risk of foodborne illness due to microbial contamination, as well as improper use of food additives, adulteration and environmental contamination. Vendors of ready-to-eat food constitute a major source of food health risk, especially through oro-faecal transmission of pathogens, and vendor related risk is particularly high in poor resource communities [3]. There are about 60,000 food vendors of ready-to-eat food in Accra, the capital city of Ghana [4]. While there is an increasing shift towards ready-to-eat food in Accra, improving the safety of such food has remained a major challenge.

A major intervention in improving the safety of ready-to-eat food or street food entails training of food vendors on hygienic handling of food [1,5]. However, this is more effective if it is evidence based, that is, if the training is based on specific data and information obtained from the trainee vendors. Quite recently, the World Health Organisation (WHO) has developed five main keys to safer food, which include keeping clean, separating raw and cooked food, cooking thoroughly, keeping food at safe temperatures, and using safe water and raw materials [6]. These five keys to safer food are of immense importance in developing countries, and equipping food vendors in such countries with such information could impact significantly on food safety. The aim of the study was to apply the WHO five keys to safer food in an evidence based training programme for food vendors to improve the safety of street food or ready-to-eat food in a poor community in Accra.

## 2. Methods

### 2.1. The Study Area

The project was carried out mainly at Chorkor, a suburb of Accra, from October, 2007 to July, 2008. The study area is located within the Dahomian ecological zone of West Africa and the vegetation is coastal savanna grassland. The climate is hot and humid and there is a bimodal rainfall pattern with a mean annual rainfall of about 1,300 mm. The mean daily temperature is 26 °C with a range of 18 °C–35 °C. The relative humidity can be as high as 97% in the mornings of wet seasons and as low as 20% in the afternoon of the dry seasons.

The study community, which is located at the outskirts of Accra, is densely populated, has a relatively high level of poverty, and is characterised by poor hygienic environments which could pose serious health risks to ready-to-eat or street food. The population of food vendors in the community is unknown, but sale of ready-to-eat food appears to be an important occupation, in addition to fishing, the main occupation.

### 2.2. Data Collection

The project involved two main phases. The first phase focused on data collection on the food handling practices of street food vendors. An attempt was made to determine the population of food vendors in the study community. This was done by collecting information from the Chief of the community and other community officials who interact with food vendors. The information collected showed that the population of food vendors in the community was about 250. Following this, a stratified method of sampling was used to statistically sample 100 food vendors of the various major food types sold, for interview using a structured questionnaire (with a Leikert scale). A total of 27 food vendors from other parts of Accra were also interviewed. The data collected focused primarily on: (i) food handling practices; (ii) environmental and personal hygiene; (iii) risk factors of oro-faecal transmission, and (iv) incidence of diarrhoea and its treatment. Additionally, data on other market factors not directly related to risk such as food types sold and market size of vendors were also collected. Personal hygiene of the vendors was assessed by their cleanliness of appearance and health, while environmental hygiene was assessed by cleanliness of the food environment, plates and other food equipment. Apart from personal and environmental hygiene, which involved data collection by observation, the other data collected were self reported by the vendors. Stool specimens were collected from all the vendors and screened for enteric pathogens of bacteria and parasites by standard microbiological methods [7–10]. For bacterial pathogens, specimens were cultured on MacConkey agar and Salmonella-Shigella agar, and the isolated organisms were identified by inoculation of various biochemical reagents. In the case of parasites, specimens were analysed microscopically.

The second phase of the project entailed a one-day risk communication workshop in which food vendors were invited to participate without incentive. At the workshop food vendors were trained in food handling using the WHO keys of safer food [6], and information synthesized from data collected in the first phase. In a highly simplified form, the vendors were also given lessons and training in various food safety related subjects, including food and personal hygiene, environment and food safety, transmission of food-borne infections, control of foodborne infections, and economic importance of food safety. The lessons presented at the workshop were developed and validated by the investigators of the study prior to the workshop. The language of communication used at the training workshop was a local dialect understood by the vendors. Following the training, data was collected from the vendors on their food handling practices after a period of six months, through a one time survey. The data was analysed and compared with data collected on food handling practices of the vendors prior to the training workshops, to help assess the impact of the training on food handling. The training impact was further evaluated by: (i) the extent to which the trained vendors shared knowledge from the workshop with others, and (ii), the assessment of the workshop by the food vendors.

### 2.3. Data Analysis

The laboratory and interview data collected from the food vendors was entered in MS-EXCEL and MS-ACCESS and analysed using SPSS to help address the objectives of the study. The strategies taken to analyse the data collected in the study, involved descriptive statistics, including geometric means, frequencies, ranges and prevalence rates of the study variables. Significant differences, associations, and interrelationships of the variables were also assessed.

### 2.4. Ethical Considerations

The protocol of the study was submitted to the Ethical Committee of The University of Ghana Medical School and consent was obtained from food vendors and the Local Authority before the interviews and training workshop.

## 3. Results

### 3.1. Demographic Characteristics of Food Vendors

The demographic characteristics of the food vendors sampled are reported as follows. Overall, the food vendors comprised 125 (98.4%) females and two (1.6%) males. The age range of the food vendors was 16–70 years with a mean age of 34 years (SD = 11.8). A total of 116 (91.3%) of the vendors were Christians, 9 (7.1%) were Moslems, while two (1.6%) did not have any religious affiliation. Overall, 33 (26%) of the food vendors did not have any form of education, 40 (22%) had Junior Secondary School Education, 28 (22%) had Primary School Education, 17 (13.4%) had Senior Secondary School Education, while nine (7.1%) had Middle School Leaving Certificate Education.

### 3.2. Prevalence of Intestinal Pathogens and Incidence of Diarrhoea among Food Vendors

No intestinal bacterial pathogen was isolated from stool specimens of any of the food vendors screened. However, a parasite, namely *Giardia lambia*, was isolated at a prevalence rate of 1% (1/100). A proportion of 12% (16) of the food vendors experienced diarrhoea every month, 15% (19) experienced it at intervals of 3-months, 4.7% (6) experienced it at intervals of 6-months, 19.7% (25) experienced it yearly, while 45.7% (58) experienced it at intervals of more than one year. A proportion of 2.4% (3) of the vendors had no experience of diarrhoea.

### 3.3. Practices, Attitudes and Perceptions of Food Vendors Related to Oro-Faecal Transmission of Intestinal Pathogens

Toilet attendance of the vendors and hand washing afterwards (prior to the training workshop) were as follows. In a day, during food sales, 69.3% (88) of the food vendors attended the toilet once for defaecation, 11% (14) attended the toilet twice, 1.6% (2) attended the toilet three times, while 18.1% (23) did not attend the toilet at all. After defaecation, 92.1% (117) *mentioned* that they always washed their hands, 3.1% (4) washed hands sometimes, while 4.7% (6) never washed hands at all. The food vendors had different modes of hand washing after defaecation, and the type of water used was portable water (pipe borne water) A total of 86.6% (110) food vendors washed their hands with water and soap, while 12.6% (16) washed hands with only water. A proportion of 71.2% (108) of the vendors discarded the water after washing, 18.2% (12) used the water for washing food equipments, while 10.6% (7) reused the water in subsequent hand washings. A high majority of 98.4% (125) of the food vendors had knowledge that some human pathogens could be transmitted by the oro-faecal route. Generally, there were no significant differences in the practices, attitudes and perceptions of the vendors based on their demographic characteristics at p < 0.05.

### 3.4. Environmental and Personal Hygiene of Food Vendors

In the assessment of environmental hygiene of the vendors, 16.5% (21) was scored as poor, 47.2% (60) was fair, while 34.6% (44) was good. In terms of personal hygiene, 4.7% (6) of the vendors had poor hygiene, 57.5% (73) had hygiene rated as fair, while 36.2% (46) had good hygiene.

### 3.5. Food Safety and Handling Practices of Food Vendors

The food handling practices of the food vendors prior to the training workshop were as follows. Before food preparation, all the food handlers washed their hands; 57% of the vendors washed hands always, 12% washed most times, while 31% did not wash often. During food preparation, 2% of the vendors admitted not using clean surfaces, while 98% used clean surfaces to various extents as follows: 47% used clean surfaces always; 17% used them most times, while 34% did not use them often. The use of separate equipment for raw and cooked food or separating the two categories of food during storage was not practiced by 1% of the vendors. On the usage of equipment, 35% of the vendors always used separate sets of equipment for raw and cooked food, 21% used separate equipment most times, while 42% did not use them frequently. On the practice of storing raw and cooked food separately, 27% of the vendors always stored raw and cooked food separately, 23% stored them separately most times, while 49% did not do this often. Pipe borne water was the water source used by 99% of the vendors for cooking and food handling, while 1% of the vendors used well water. A proportion of 81.9% of the food vendors stored food overnight and sold to consumers, 55.9% used refrigeration facilities for storage, while 15.7% kept food uncovered. In a day, one food vendor sold food to an average of 117 people ranging from 1–500 people. A wide range of ready-to-eat food was sold by the vendors (Table 1). Generally, there were no significant differences in the food handling practices of vendors based on their demographic characteristics at p < 0.05.

### 3.6. Training of Food Vendors

Overall, a total of 100 food vendors (including the majority of those who took part in the pre-workshop survey) were trained in various aspects of food safety. There was also an interactive phase of the workshop, during which the food vendors asked pertinent questions related to food safety and suggested ways the safety of ready-to-eat food could be improved. This actually enriched the workshop, and made knowledge transfer to the vendors easier. Collecting and analysing stool specimens from the vendors before the training was useful because it helped to inform the vendors practically about oro-faecal transmission, and the possible role of food vendors in the transmission process. Table 2 shows the self-reported food handling practices of the vendors before and after the training workshop, as well as the overall impact of the training. Impact assessment of the training showed quite a favourable outcome: overall, 67.6% of the food vendors had acquired some knowledge and were putting it into practice; 27% had also acquired some knowledge but were not practicing it; while 5.4% indicated not acquiring any knowledge.

A proportion of 42% of the food vendors indicated that they had shared the knowledge they acquired from the training workshop with other people. Assessment based on specific indicators related to the WHO food safety keys showed the following: the rate of washing hands always before and during food preparation improved from 57% to 100%. Separation of raw and cooked food always during storage and using separate sets of equipment for them was practiced by 33.3% of the vendors after the workshop. However, before the workshop, 13.4% of the vendors used separate equipment for the two types of food while 91.3% separated the two types of food during storage. Keeping food at safe temperature was assessed only after the workshop, and showed that 27% of the vendors always kept food in the refrigerator if stored overnight, while 59.5% always reheated cooked food before selling.

## 4. Discussion and Policy Implication

Street food is known to pose significant risk to consumers and street food handlers constitute an important factor in improving the safety of street food. In this study, we monitored the handling practices and risk factors among food vendors with the aim of utilizing the information to institute corrective measures through an evidence-based training programme. The prevalence of intestinal/diarrhoeal pathogens among the vendors was low (1%). However, the incidence of diarrhoea among the vendors was 51.4% annually. Incidence rate of a disease is known to provide a better measure of true frequency in a population compared to the prevalence rate [11]. The incidence rates of diarrhoea among the food vendors indicates that food vendors in the study area constitute an important source of intestinal pathogens and could therefore be important agents of oro-faecal transmission in these communities. In this study we isolated only *Giardia lambia* from one food vendor. However, a wide range of intestinal pathogens have been isolated from street food vendors, and include pathogens such as *Salmonella typhi*, non typhoidal salmonellae, *Entaemoeba histolytica*, *Ascaris lumbricoides*, *Enterobius vermicularis, Trichuris trichiura* [12–15]. Carriage of intestinal pathogens by food vendors could result in widespread transmission of the pathogens in a community, especially in countries like Ghana where food vendors form a significant part of the food supply chain. Apart from the vendors being an important source of enteric pathogens, high prevalence of food vendors who did not take any medication for diarrhoea or practiced self medication is a cause for concern, as such vendors could carry pathogens for a long period and pose risk to consumers

A majority of the food vendors attended the toilet during selling period and the standard practice of washing hands with water and soap after toilet was done by 98%. This is quite encouraging and may be due to the fact that a high majority of the vendors (98.4%) had knowledge on oro-faecal transmission of pathogens. However, a proportion of 28.8% did not discard water after the toilet related hand washing, but used it for cleaning food equipment or for subsequent hand washing. This practice could encourage dissemination of pathogens in the food environment with possible outbreaks [3,16,17]. Assessment of personal and environmental hygiene of the vendors showed that 4.7% and 16.5% of the food vendors had poor personal and environmental hygiene, respectively. Thus generally, personal and environmental hygiene of the vendors were good which may be due to the increasing awareness of food safety among the vendors as well as the inspection of vendoring sites by sanitation officers. Similar findings have also been reported [18].

With the aid of a questionnaire, we assessed compliance with the WHO five keys to safer food among the vendors, which was based mainly on self reported information by the vendors. Though, generally, compliance with the various safer food keys was high, a small proportion of the vendors constantly utilized the food safety keys. For example, overall, 99% of the vendors stored raw and cooked food separately, however, only 27% practiced this always. The WHO five keys to safer food are recognised as a standard way of producing and maintaining safe food in communities. Maximum operation of these food safety keys ensures consumer protection against food health hazards. In this study, because the food vendors do not always comply with the food safety keys, the safety of the street food sold by the vendors cannot be guaranteed. We did not sample street food sold by the vendors for investigation but studies on the food have reported high microbial counts and the presence of a number of pathogens [18–22].

In this study which was carried out in Accra, the capital city of Ghana, an average of 117 people bought food from one food vendor in a day. In Latin America, street food purchases account for up to 30% of urban household spending, while in countries like Bangkok, 20,000 street food vendors provide city residents with an estimated 40 percent of their overall energy intake [23]. Generally, about 2.5 billion people worldwide patronise street food [23]. The relationship between street food and the general population brings to focus safety considerations, and interventions such as regular health education of food vendors are urgently required to protect public health.

The training workshop organised for the food vendors produced some significant results though food handling practices of the vendors had not improved perfectly. Overall, 67.6% of the vendors had acquired some knowledge from the workshop and were putting it into practice. Washing hands always during food preparation increased from a rate of 57% to 100% after the training. However, significant inprovements were not observed for the other WHO safer food keys tested especially, using separate utensils for raw and cooked food, including separating the two food types during storage, and storing food overnight in the refrigerator. Employment of these food safety keys requires food utensils and other equipment such as refrigerators. The study was carried out in a poor resource community and is likely lack of resources may have accounted for poor performance of the vendors with regard to these food safety keys despite the significant impact of the training.

There are some limitations of this study. Firstly, we surveyed different groups of vendors before and after the training workshop. Though the vendors came from the same community, this may introduce some bias in assessing the effectiveness of the training workshop on behavioral change. A second limitation of the study is that a small sample size of 37 vendors completed the follow-up survey. The workshop was carried out six months after the study; some vendors may have moved from the location or changed trade, making follow up difficult. A third limitation of the study is that most of the data we collected from the vendors were self reported by the vendors, which may also introduce some bias in the behaviors of the vendors studied.

It is concluded that generally, food vendors have information on food safety such as hygiene and disease prevention. However, they require an impulse such as a training workshop to put knowledge on food safety into practice. Lack of facilities among food vendors in poor resource communities could be a major constraint to the employment of good food safety practices.

## Figures and Tables

**Table 1.** Types of ready-to-eat food sold by the vendors.

**Food**	**Description**	**Mode of Cooking**

Koko (porridge)	Fermented maize dough	Boiling
Koose	Fried bean cake	Frying
Kenkey	Fermented maize dough	Wrapped in maize husks and boiled
Banku	fermented maize dough dumpling	Heating with continous stirring
Rice	Boiled rice	Boiling
Wankye	Rice and beans	Boiling
Fufu	Cassava with plantain/cocoyam	Boiling and pounding
Omutuo	Boiled rice dumpling	Boiling and stirring
Beans	Cooked beans	Boiling
Okra soup	Okra and other vegetables; palm oil added	Boiling
Yam and plantain	Boiled or fried	Boiling or frying
Chofi	Turkey meat	Fried
Salad	Mixture of leafy vegetables	No cooking

**Table 2.** Impact of training and self-reported food handling practices of vendors, before and after the training workshop.

**Parameter**	**Pre-training (%)**	**Post-training (%)**
**Hand washing before food preparation**		
Always done	57	100
Most times done	12	0
Not often done	31	0
Not done	0	0
**Use of separate equipments for cooked and raw food**		
Always done	35	35.1
Most times done	21	16.2
Not often done	42	45.9
Not done	1	2.7
**Refrigeration of stored cooked food**		
Always done	n/e	27
Most times done	n/e	16.2
Not often done	n/e	54.1
Not done	45.1	2.7
**Reheating of stored cooked food**		
Always done	n/e	59.5
Most times done	n/e	8.1
Not often done	n/e	29.7
Not done	n/e	2.7
**Sharing training experience with others**		
Yes	n/a	48.6
No	n/a	51.4
**Overall impact of training workshop**		
Knowledge acquired and practicing	n/a	67.6
Knowledge acquired but not practicing	n/a	27
No knowledge acquired	n/a	5.4

Note: n/e indicates not evaluated; n/a indicates not applicable;

Data on post training food handling practices is based on 37 vendors;

No significant differences in the food handling practices of vendors related to their demographic characteristics were observed at p < 0.05.
